# A Wideband Circularly Polarized Antenna with a Multiple-Circular-Sector Dielectric Resonator

**DOI:** 10.3390/s16111849

**Published:** 2016-11-03

**Authors:** Son Trinh-Van, Youngoo Yang, Kang-Yoon Lee, Keum Cheol Hwang

**Affiliations:** School of Electronic and Electrical Engineering, Sungkyunkwan University, Suwon 440-746, Korea; jsonbkhn@gmail.com (S.T.-V.); yang09@skku.edu (Y.Y.); klee@skku.edu (K.-Y.L.)

**Keywords:** aperture-coupled feeding, circular polarization, dielectric resonator antenna, multiple-circular-sector structure, wide bandwidth

## Abstract

This paper presents the design of a wideband circularly polarized antenna using a multiple-circular-sector dielectric resonator (DR). The DR is composed of twelve circular-sector DRs with identical central angles of 30${}^{\circ }$ but with different radii. A genetic algorithm is utilized to optimize the radii of the twelve circular-sector DRs to realize wideband circular polarization. The proposed antenna is excited using an aperture-coupled feeding technique through a narrow rectangular slot etched onto the ground plane. An antenna prototype is experimentally verified. The measured −10 dB reflection and 3 dB axial ratio (AR) bandwidths are 31.39% (1.88–2.58 GHz) and 19.30% (2.06–2.50 GHz), respectively, covering the operating bands of the following systems: UMTS-2100 (2.145 GHz), WiMAX (2.3 GHz), and Wi-Fi (2.445 GHz). A measured peak gain of 7.65 dBic at 2.225 GHz and gain variation of less than 2.70 dBic within the measured 3 dB AR bandwidth are achieved. In addition, the radiation patterns of the proposed antenna are presented and discussed.

## 1. Introduction

With the rapid development of wireless communication systems, the demand for high efficiency, wideband, and compact antennas has increased significantly [1,2]. Various types of antennas, such as microstrip patch antennas [3,4] and dielectric resonator antennas (DRAs) [5,6], have been investigated and developed for wireless communications. However, microstrip patch antennas (known as metallic-based antennas) usually have low radiation efficiency levels, owing to conductor loss. Meanwhile, DRAs, with no conductor loss, offer the advantages of high radiation efficiency, ease of excitation, small size, and wide bandwidth [7,8]. Therefore, DRAs have become an excellent candidate for use in wireless communication applications.

DRAs with a diversity of antenna geometries and feeding schemes have been designed for both linearly polarized (LP) and circularly polarized (CP) systems. Compared to LP antennas, CP antennas are more feasible, because they can reduce the losses caused by propagation effects and misalignment between the transmitting and receiving antennas [9]. Thus, the designs of CP DRAs have received much attention recently [10,11,12,13,14,15,16]. The technique for realizing circular polarization through a DRA mostly requires either modifying the excitation mechanism or using a specially-shaped dielectric resonator (DR). CP DRAs with different excitation techniques to excite simple regularly-shaped DRs have been reported. For example, in one study [10], Sulaiman et al. proposed a rectangular DRA excited by concentric open half loops to achieve a 3 dB axial ratio (AR) bandwidth of 14%. Zou et al. introduced a CP DRA fed by a lumped resistively loaded monofilar-spiral slot, resulting in an AR bandwidth of 18.7% [11]. A modified cross-slot design was also utilized to excite a rectangular DRA to produce circular polarization with an AR bandwidth of 24.6% [12]. Meanwhile, designs of CP DRAs with specially-shaped DRs fed by the aperture-coupled feeding technique were also investigated. In one such study [13], an AR bandwidth of 11.57% was achieved using a Spidron fractal-shaped DR. A CP DRA with a grooved rectangular DR was also presented, reportedly showing an AR bandwidth of 23.75% [14]. Pan et al. introduced a trapezoidal DRA [15] which offered an AR bandwidth of 21.5%. Recently, a CP DRA with an AR bandwidth of 18.2% was realized in a design which used two rectangular DR layers stacked together at a rotating angle relative to adjacent layers [16].

In this paper, we propose a wideband CP DRA which uses a specially-shaped DR, referred to as a multiple-circular-sector DR. The cylindrical DR was divided into twelve circular-sector DRs having identical central angles of 30${}^{\circ }$. Circular polarization is achieved in this design by optimizing the radii of the twelve circular-sector DRs using a genetic algorithm (GA), a technique which has been applied to solve many antenna optimization problems [17,18]. As a result, the proposed antenna exhibits wideband CP characteristics. The proposed antenna was excited by the coupling between a narrow rectangular slot etched on the ground plane and a 50-Ω microstrip feeding line located underneath the slot. A simulation was carried out using the ANSYS High-Frequency Structure Simulator (HFSS). An antenna prototype with optimal design parameters was fabricated and tested. This paper is organized as follows. Section 2 introduces the design concept of the proposed antenna. The experimental results and a comparison between measured and simulated results are presented in Section 3. Finally, the conclusion is given in Section 4.

## 2. Antenna Design

**Figure 1:**
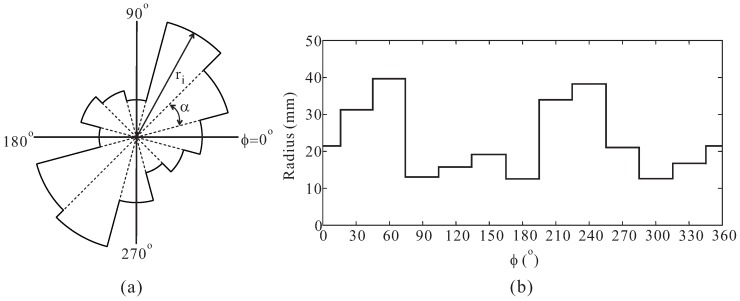
Multiple-circular-sector structure: (**a**) Shape of the multiple-circular-sector; (**b**) Radii at different angles.

Figure 1 illustrates the concept of the multiple-circular-sector structure. The multiple-circular-sector structure was previously introduced in earlier works [19,20]. It was initially proposed by Yeung et al. for the design of an ultra-wideband patch antenna [19]. In a subsequent work [20], Yeung et al. successfully implemented a wideband circularly polarized multiple-circular-sector slot antenna. As shown in Figure 1a, the multiple-circular-sector shape includes twelve small circular sectors, all with identical central angles of *α* = 30${}^{\circ }$. Thus, these circular sectors create a complete circular shape of 360${}^{\circ }$. Each circular sector has a different radius $r_{i}$ (*i* = 1 to 12). The relationship between the radius and angle *ϕ* is given in Figure 1b.

**Figure 2:**
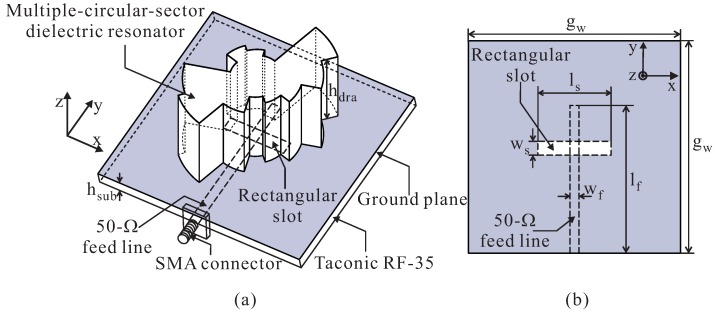
The geometry of the proposed antenna: (**a**) 3D view; (**b**) Feeding configuration. SMA: SubMiniature version A.

In this study, the DR is designed using the concept of a multiple-circular-sector shape. It is termed the multiple-circular-sector DR. Figure 2a shows the geometry of the proposed antenna. The feeding structure is illustrated in Figure 2b. The proposed antenna includes a multiple-circular-sector DR, a ground plane, a 50-Ω microstrip feed line, and a Taconic RF-35 dielectric substrate (with a dielectric constant of 3.5, a thickness of $h_{sub}$ = 1.52 mm, and a loss tangent of 0.0018). The 50-Ω microstrip feed line with a length of $l_{f}$ and a width of $w_{f}$ is printed on the bottom layer of the dielectric substrate. The ground plane, which is mounted on the upper layer of the dielectric substrate, has dimensions of $g_{w}$ × $g_{w}$. A narrow rectangular slot with dimensions of $w_{s}$ × $l_{s}$ is etched onto the ground plane. The energy from the microstrip feed line is coupled through this narrow slot to excite the proposed DR. The multiple-circular-sector DR with a dielectric constant of 9.8 is composed of twelve circular-sector DRs, each with an identical central angle of 30${}^{\circ }$ and different radii. The GA is applied to optimize the radii of the twelve circular-sector DRs to achieve wideband CP operation. In the implementation of the GA, an iteration number of 1000, a population of 20, a mutation rate of 0.1, and single-point crossover scheme are used. The optimal design parameters of the proposed antenna are summarized in Table 1 for the generation of left-handed circular polarization (LHCP).

**Figure 3:**
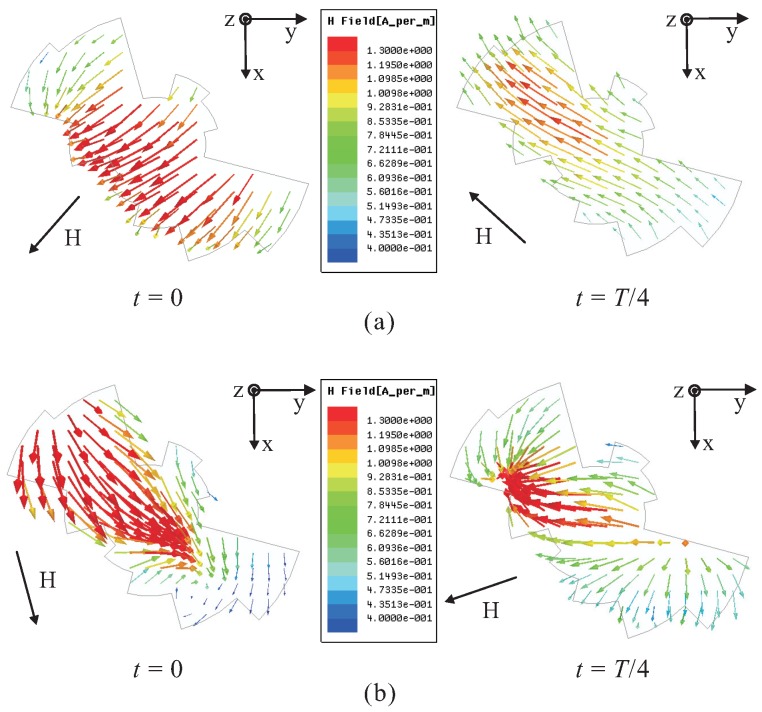
Simulated H-field distributions on the top surface of the proposed multiple-circular-sector dielectric resonator (DR) with time period *T* at: (**a**) 2.3 GHz; (**b**) 2.5 GHz.

In order to verify the generation of LHCP, the H-field distribution on the top surface of the proposed multiple-circular-sector DR is investigated. Figure 3a presents the simulated H-field distribution observed in the positive *z*-direction at a frequency of 2.3 GHz. When *t* = 0, the vector of the major H-field distributions points from the upper right corner to the lower left corner. In contrast, the vector of the major H-field distributions at *t* = *T*/4 points from the lower right corner to the upper left corner. This vector is orthogonal to that at *t* = 0, and rotates clockwise as the time *t* elapses, thereby generating LHCP in the positive *z*-direction. The H-field distribution observed in the positive *z*-direction at 2.5 GHz is illustrated in Figure 3b. Similar to the case at 2.3 GHz, the vector of the major H-field distributions at *t* = 0 is orthogonal to that at *t* = *T*/4, and rotates clockwise as the time *t* increases. Therefore, LHCP is also produced in the positive *z*-direction.

**Figure 4:**
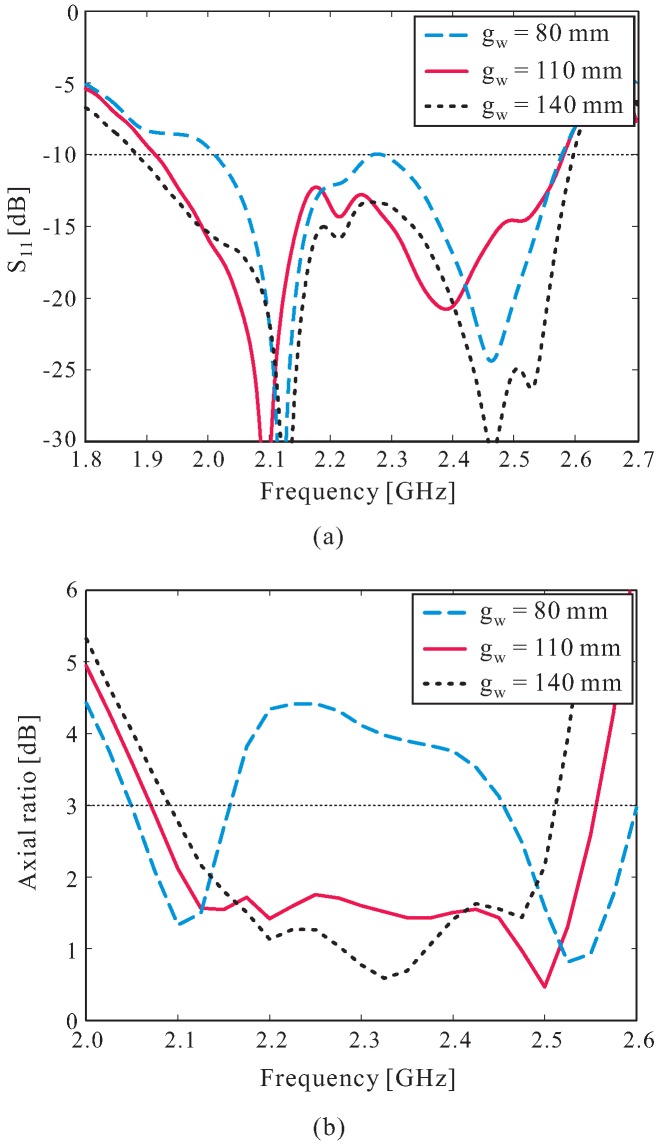
Effect of the ground plane size $g_{w}$ on: (**a**) Reflection coefficient; (**b**) Axial ratio.

The effects of the ground plane size $g_{w}$ on the reflection coefficient and AR performance are also investigated. The simulated results are illustrated in Figure 4, in which the AR values are observed in the broadside direction (*θ* = 0${}^{\circ }$). As shown in the figure, dual-band CP operation is achieved with $g_{w}$ = 80 mm. When $g_{w}$ increases, two CP bands are merged to form a wide 3 dB AR bandwidth. In addition, the reflection coefficient in the middle frequency range is improved, while the overall −10 dB reflection bandwidth is only slightly affected when increasing the value of $g_{w}$. However, the overall 3 dB AR bandwidth of the proposed antenna is reduced when $g_{w}$ is increased further. The value of $g_{w}$ is finally set to 110 mm, as this value provides the widest 3 dB AR bandwidth.

**Figure 5:**
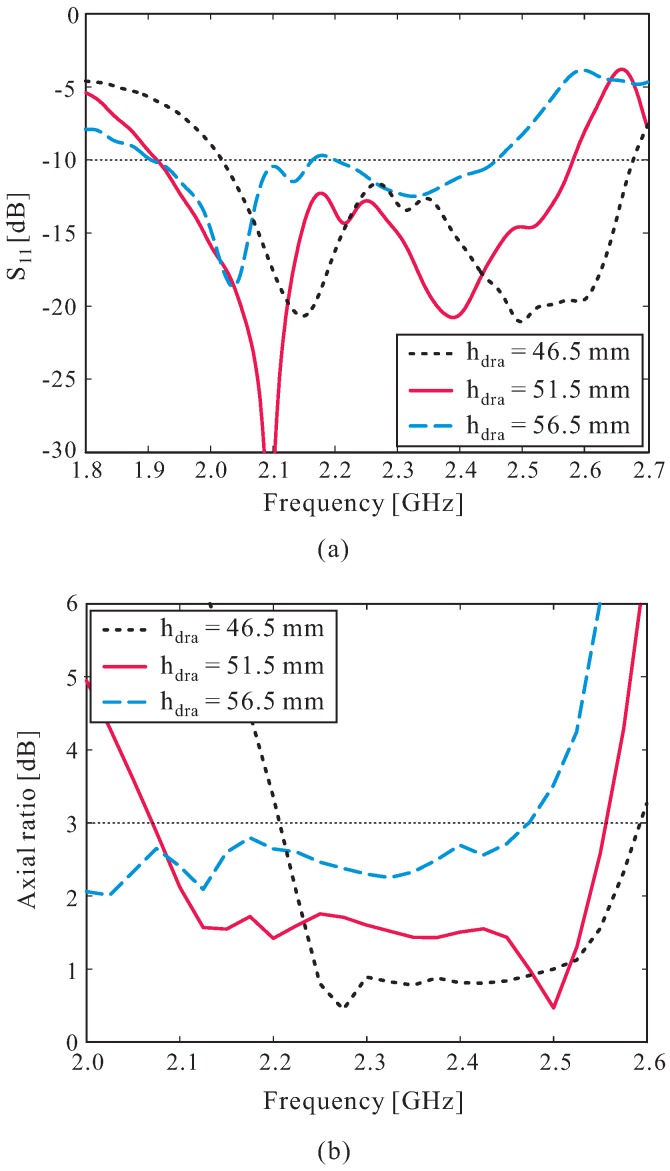
Effect of the DR height $h_{dra}$ on: (**a**) Reflection coefficient; (**b**) Axial ratio.

The effects of the DR height $h_{dra}$ on the reflection coefficient and AR performance were studied, and the simulated results are illustrated in Figure 5. It was found that the operating frequency band moves to a lower frequency region, and that the AR levels are increased close to 3 dB when $h_{dra}$ is increased. When $h_{dra}$ equals 56.5 mm, the reflection coefficient levels around 2.18 GHz are increased to more than −10 dB, resulting in two separate −10 dB reflection bandwidths (see Figure 5a). Meanwhile, as shown in Figure 5b, the 3 dB AR bandwidth becomes narrower when $h_{dra}$ is reduced to less than 46.5 mm. Finally, the optimal value of $h_{dra}$ was found to be 51.5 mm.

**Figure 6:**
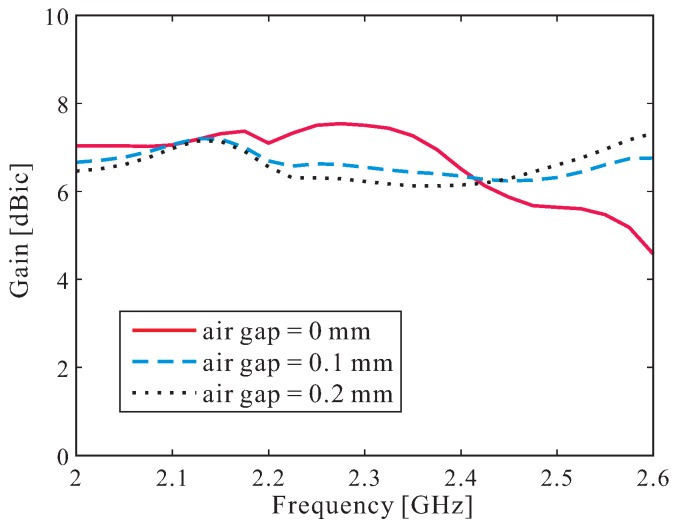
Effect of an air gap on antenna gain.

Finally, we conducted a simulation to investigate the effect of a slight air gap between the DR layer and the ground plane. The simulated results are shown in Figure 6. It is evident that the air gap affects the antenna gain. Owing to the existence of a slight air gap between the DR layer and the ground plane during the gluing process, as well as the fabrication tolerances, a maximum gain tolerance of 1.4 dB is observed when air gap varies from 0 to 0.2 mm.

The design guideline for the proposed CP DRA with a multiple-circular-sector DR is as follows. First, choose initial design parameters for the coupling slot width $w_{s}$, the length $l_{s}$, and the microstrip stub length ($l_{f}$ − $g_{w}$/2) based on earlier work [21]. In this work, the initial values of slot length $l_{s}$, slot width $w_{s}$, and microstrip stub length ($l_{f}$ − $g_{w}$/2) are set at 0.25$\lambda_{0}$, 0.2$l_{s}$, and 0.25$\lambda_{g}$, respectively, where $\lambda_{0}$ is the free-space wavelength and $\lambda_{g}$ is the guided wavelength in the dielectric substrate corresponding to the designed frequency of 2.3 GHz. The ground plane size $g_{w}$ is initially set at $\lambda_{0}$. Next, obtain a wide bandwidth of −10 dB reflection and a bandwidth of 3 dB AR by optimizing the radii of the twelve circular-sector DRs. Finally, adjust the parameters $w_{s}$, $l_{s}$, $g_{w}$, $h_{dra}$, and $l_{f}$ to improve the reflection coefficient and AR performance further.

## 3. Experimental Results and Discussion

**Figure 7:**
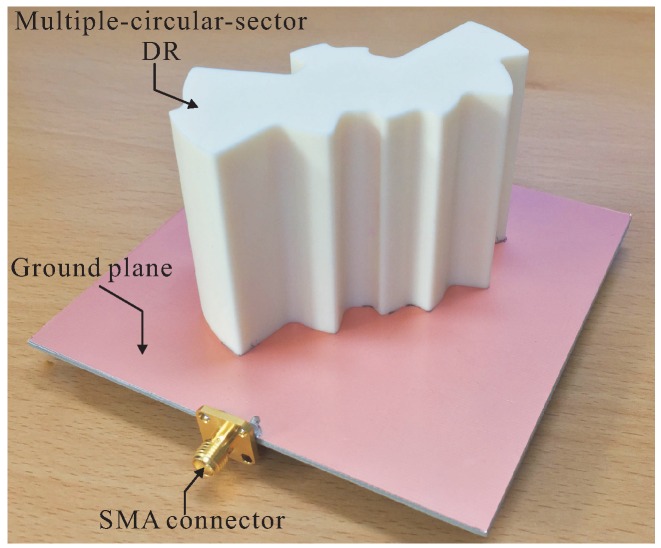
Photograph of the fabricated antenna.

**Figure 8:**
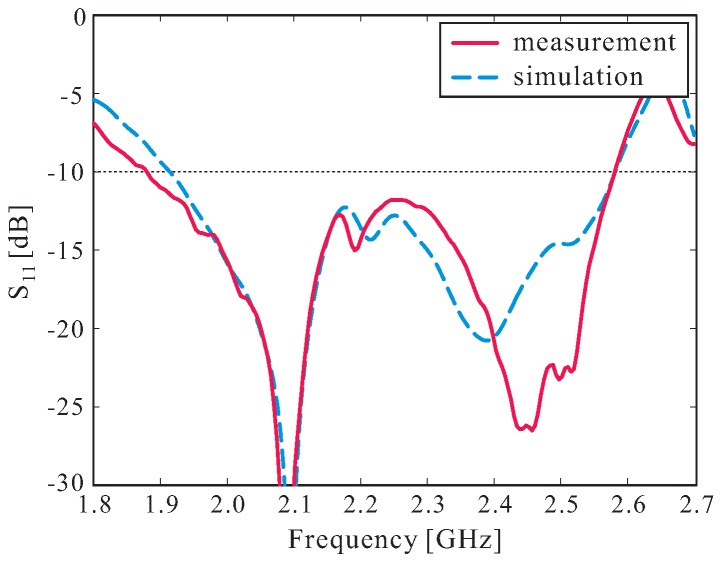
Measured and simulated reflection coefficients.

Based on the optimized design parameters listed in Table 1, a prototype of the proposed antenna was fabricated. A 99.5% alumina ceramic material with a dielectric constant of 9.8 and a loss tangent of 0.0001 was utilized to fabricate the proposed DR. Figure 7 shows a photograph of the fabricated antenna. Epoxy adhesive was used to glue the bottom layer of the multiple-circular-sector DR to the ground plane. An Agilent 8510C network analyzer was used to measure the reflection coefficient of the fabricated antenna. The measured and simulated results of the reflection coefficients are illustrated in Figure 8. Good agreement between the measurement and simulation results was achieved. It was found that the measured and simulated −10 dB reflection bandwidths were 31.39% (1.88–2.58 GHz) and 29.33% (1.91–2.58 GHz), respectively.

**Figure 9:**
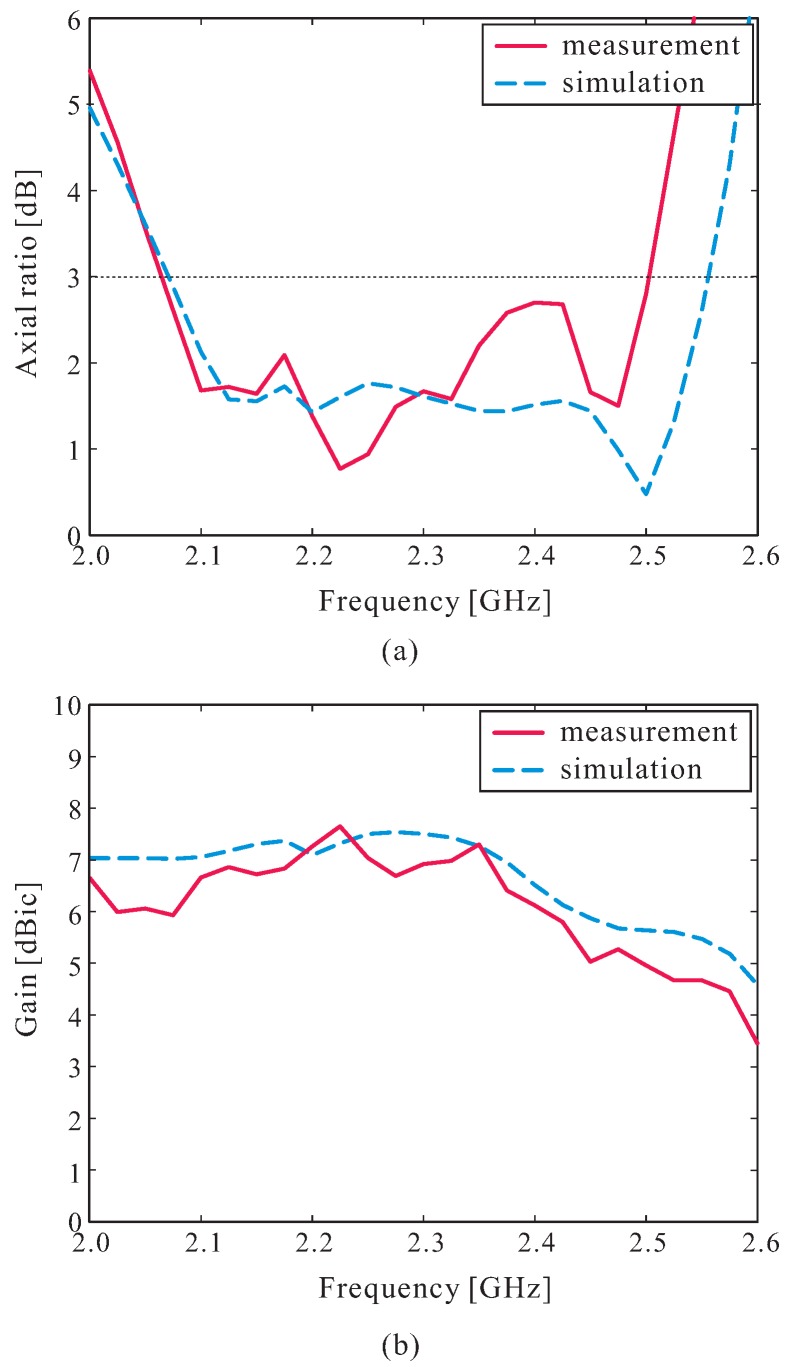
Measured and simulated results of: (**a**) Axial ratios; (**b**) Left-handed circular polarization (LHCP) gains.

For the measurement of the radiation characteristics of the proposed antenna, a dual-polarized rectangular horn antenna in a RF anechoic chamber was utilized. The measured and simulated results of the ARs and LHCP gains of the proposed antenna in the broadside direction (*θ* = 0${}^{\circ }$) are shown in Figure 9. As can be observed in Figure 9a, the measured and simulated 3 dB AR bandwidths were 19.30% (2.06–2.50 GHz) and 21.17% (2.07–2.56 GHz), respectively. Within the measured 3 dB AR bandwidth, a measured peak LHCP gain of 7.65 dBic at 2.225 GHz and gain variation of less than 2.70 dBic were achieved, as shown in Figure 9b. Reasonable agreement was noted between the measured and simulated results, with some discrepancies mainly attributed to experimental tolerances, fabrication imperfections, and the existence of a slight air gap between the DR layer and the ground plane during the gluing process, as discussed in Figure 6.

**Figure 10:**
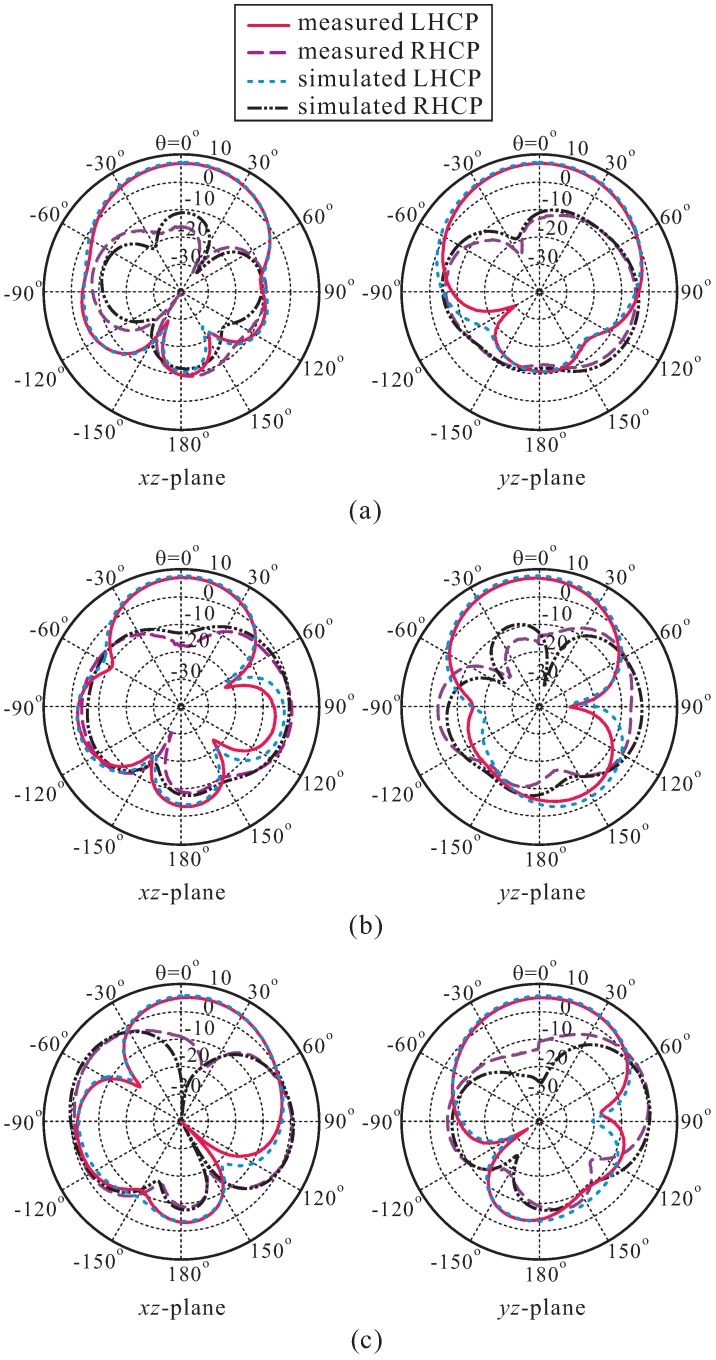
Measured and simulated radiation patterns of the proposed antenna at: (**a**) 2.1 GHz; (**b**) 2.3 GHz; (**c**) 2.5 GHz. RHCP: Right-handed circular polarization.

Figure 10 plots the measured and simulated radiation patterns of the proposed antenna on the *xz*-plane (*ϕ* = 0${}^{\circ }$) and *yz*-plane (*ϕ* = 90${}^{\circ }$) at the three frequencies of 2.1 GHz, 2.3 GHz, and 2.5 GHz. It is evident that the proposed antenna is an LHCP antenna, and that the radiation patterns are directional toward the broadside direction. In addition, on both planes, the LHCP gains are 20.30 dB, 20.38 dB, and 14.50 dB higher than the right-handed circular polarization (RHCP) gains in the broadside direction at these three corresponding frequencies. The deterioration of the radiation patterns is due to the asymmetry of the proposed DR configuration along the x-axis and y-axis. This effect becomes stronger at higher frequencies.

In order to evaluate the performance of the antenna, a comparison of the proposed antenna and antennas presented in earlier works [11,12,13,14,15] was performed. These results are illustrated in Table 2. It is evident that the proposed antenna exhibits a wider 3 dB AR bandwidth and a higher peak gain compared to the earlier antennas [11,13]. Two antennas [12,14] produced a wider 3 dB AR bandwidths, but their peak gains were lower than that of the proposed antenna. Another antenna [15] employed a trapezoidal DR with overall dimensions of 1.16$\lambda_{0}$ × 1.16$\lambda_{0}$ × 0.44$\lambda_{0}$ ($\lambda_{0}$ is the wavelength corresponding to the center frequency of the AR band). Meanwhile, the overall dimensions of the proposed antenna were only 0.84$\lambda_{0}$ × 0.84$\lambda_{0}$ × 0.39$\lambda_{0}$. Therefore, compared to antennas presented in earlier works, the proposed antenna exhibits good performance in terms of antenna gain and 3 dB AR bandwidth, as well as small size.

## 4. Conclusions

The design of a microstrip-fed wideband CP antenna using a multiple-circular-sector DR was proposed, fabricated, and measured. The DR was composed of twelve circular-sector DRs, all with identical central angles of 30${}^{\circ }$, but with different radii. The radii were optimized using a GA to realize wideband CP operation. The experimental results proved that the proposed antenna has a wide −10 dB reflection bandwidth of 31.39% (1.88–2.58 GHz), a wide 3 dB AR bandwidth of 19.30% (2.06–2.50 GHz), a peak gain of 7.65 dBic, and a high level of LHCP gain compared to the RHCP gain in the broadside direction. The CP operation frequency range of the proposed antenna covers the operating bands of several systems, such as UMTS-2100 (2.145 GHz), WiMAX (2.3 GHz), and Wi-Fi (2.445 GHz). Therefore, the proposed antenna is feasible for use as a wideband CP antenna element for wireless communication applications and satellite communication systems.

## Figures and Tables

**Table 1:** The optimized design parameters of the proposed antenna.

Parameter	Value	Parameter	Value
$r_{1}$	22.21 mm	$r_{11}$	12.41 mm
$r_{2}$	32.04 mm	$r_{12}$	16.51 mm
$r_{3}$	40.25 mm	$g_{w}$	110 mm
$r_{4}$	12.70 mm	$w_{s}$	7.50 mm
$r_{5}$	16.26 mm	$l_{s}$	38.00 mm
$r_{6}$	19.39 mm	$w_{f}$	3.50 mm
$r_{7}$	12.60 mm	$l_{f}$	76.50 mm
$r_{8}$	34.90 mm	$h_{dra}$	51.50 mm
$r_{9}$	38.28 mm	$h_{sub}$	1.52 mm
$r_{10}$	22.10 mm		

**Table 2:** Comparison of the proposed antenna with antennas in previous studies. Note that $\lambda_{0}$ is the wavelength corresponding to the center frequency of the AR band. CP: circularly polarized; DRA: Dielectric resonator antenna.

Structure	Description	−10 dB Reflection Bandwidth (GHz)	3 dB AR Bandwidth (GHz)	Dimensions ($\lambda_{0}^{3}$)	Peak Gain (dBic)
[11]	CP DRA fed by a lumped resistively loaded monofilar-spiral-slot	1.86–3.22 (53.5%)	2.26–2.72 (18.7%)	0.36 × 0.36 × 0.083	2.0–5.0
[12]	CP DRA fed by a modified cross-slot	2.19–2.92 (28.6%)	2.25–2.88 (24.6%)	0.43 × 0.43 × 0.27	4.3–6.1
[13]	CP DRA with a Spidron fractal DR	4.32–6.30 (37.29%)	5.13–5.76 (11.57%)	0.91 × 0.91 × 0.13	2.20–3.16
[14]	CP DRA with a grooved rectangular DR	1.94–2.92 (40.33%)	2.30–2.92 (23.75%)	0.35 × 0.35 × 0.086	2.30–4.23
[15]	CP DRA with a trapezoidal DR	2.88–4.04 (33.5%)	3.11–3.86 (21.5%)	1.16 × 1.16 × 0.44	5.28–8.39
Proposed antenna	CP DRA with a multiple-circular-sector DR	1.88–2.58 (31.39%)	2.06–2.50 (19.30%)	0.84 × 0.84 × 0.39	4.95–7.65
